# Degeneration of the Teeth

**Published:** 1883-04

**Authors:** H. Cowe


### Degeneration of the Teeth.

BY H. COWE, D.D. S.

(Read before the Michigan State Dental Society.)

It is only a few years, comparatively, since dentistry was in its infancy. None of the improvements we now have were in existence, nor was there the progress in dental education that has since been attained; indeed, the greatest change has been made in the memory of those now in active practice; the improvements have followed so closely upon one another that one has hardly time to examine and test their value as a permanency, before they are followed by something else supposed to be better. Yet, in despite of this constant change and supposed progress, teeth decay and are lost, and each succeeding generation seems to have, if possible, worse teeth than the preceding. We will try to show in this paper that this is the case, and try, if possible, to show the causes that are at work in producing them.

Having no dental data or record to give us any positive light—at least none that has come under my notice—I will therefore have to resort to personal experience and observation, and in doing this I wish to recall to the minds of those present their own; for no one who has been operating for any length of time can have failed, I think, to have noted the fact that the teeth of children of to-day are more soft and more predisposed to decay than were the teeth of their parents when at the same age, judging from the condition we find them in at adult life. My retrospect can only extend over twenty years; but having in that time seen and attended to many persons of all ages, can compare at least three generations, and can speak of what may be observed in so short a time. There cannot be a very great contrast afforded in so limited a period, but we can see the children will resemble the parents, and have reproduced all of the little peculiarities that are hereditary. We will also see if my observation be correct, that while the family features are preserved, that the physical health and power of resisting disease in the teeth is not.

The teeth of children now, as compared with those of their parents, are more porous, and that they fail to become so dense and hard as they become older, as we had been taught would be the case; and it does not seem as though any length of years could fill up the large cells and produce the density of structure to be found in those of their ancestors.

Only a very few years since, and the dentist was by the masses almost unknown. Even here in Detroit, when a boy, thirty years ago or more, I was sent by my parents to our family doctor when I needed anything done to my mouth; and he, when he learned my errand, would seat me on the floor, ask me which one of my teeth caused the trouble, and then proceed to relieve me of my suffering, and at the same time of the tooth. This may be, I think, the case with some of those now before me; for though we had several good dentists at that time here, yet, except by the comparatively few, they were not known or employed. The value of teeth was not taught. Cleaning the teeth not so generally practiced as now, and the teeth were often used for improper purposes, such, for example, as nut-crackers; and decay, once established, the tooth was lost in time without the effort to save them that is now so strongly made. Very few, comparatively, wore artificial teeth, because teeth were not so generally lost as at this time.

Now, when every little village has one or more dentists, and the large cities are full of them; when attention is so generally aroused to the importance of healthy teeth, we might conclude that teeth would be but seldom lost; that though attacked by disease dental skill would arrest the decay. Is such a result generally the rule—and why is it not? We think it is because of the difference in the structure and general make-up of the teeth themselves. We base our belief in this conclusion upon the fact before stated that teeth of this generation are not so strong, well formed, nor so dense as those of the past.

Soft teeth, as we all know, cut very easily, are much more liable to decay, and when they do begin to go, go very fast, so that oftentimes the cavity will appear very small, only a mere seam in many cases, but when broken into found burrowed to the very center of the tooth with decay; a soft pulpy mass of disorganized bone, looking much like tooth substance that has been deprived of its phosphates by the action of acid.

There seems too to be some difference between the teeth of the sexes; the boys' teeth are, as a rule, better formed, jaws larger than those of the girls.

They seem to require much less attention from the dentist, and the male adult has less often artificial teeth than the female of the same age. Now, this is not due to care on the part of the boy, but because the tooth substance is better. Natives of this country seem to have much more irregularity in the teeth and jaws. Large teeth and jaws, with full dentures, are to be found more frequently in the mouths of foreigners. Very few born here attain the age of fifteen without losing more or less of their teeth, unless they have been protected by fillings.

Absence of the phosphates seems to be the reason why the teeth of the present day are to be found so frail that decay can be arrested only for a time. Now, if this be true, and this structural difference exists, it becomes a question of the first importance to discover the cause and its cure.

If we used more the coarse cereals, and not altogether the results of the perfect milling we do, lived in better ventilated rooms, had abundant exercise, we would be more healthy, and would have teeth that could tear tough beef and cut and masticate hard crust.

Climate, surely, is not the cause of the early and rapid decay of the teeth as we find them at the present time, for the Indians, until injured by the habits of civilization, possessed perfect teeth, so far as we know, and if they developed perfect physical frames and teeth almost free from disease surely the climate is not at fault for our lack in this respect, but we must attribute it to the vices and defects of our civilization.

